# Equine Encephalomyelitis Outbreak, Uruguay, 2023–2024

**DOI:** 10.3201/eid3101.240915

**Published:** 2025-01

**Authors:** Sandra Frabasile, Noelia Morel, Ramiro Pérez, Lucía Moreira Marrero, Analia Burgueño, María Noel Cortinas, Lucía Bassetti, Raúl Negro, Sirley Rodríguez, Victoria Bórmida, Valeria Gayo, Victor Costa de Souza, Felipe Gomes Naveca, Mariela Martínez Gómez, Lionel Gresh, Jairo Mendez-Rico, Héctor Chiparelli, Adriana Delfraro

**Affiliations:** Universidad de la República, Montevideo, Uruguay (S. Frabasile, L. Moreira Marrero, A. Delfraro); Ministerio de Salud Pública, Montevideo (N. Morel, A. Burgueño, M.N. Cortinas, V. Bórmida, H. Chiparelli); Ministerio de Ganadería Agricultura y Pesca, Montevideo (R. Pérez, L. Bassetti, R. Negro, S. Rodríguez, V. Gayo); Instituto Leônidas e Maria Deane, Manaus, Brazil (V. Costa de Souza, F.G. Naveca); Instituto Oswaldo Cruz, Rio de Janeiro, Brazil (F.G. Naveca); Pan American Health Organization, Washington, DC, USA (M. Martínez Gómez, L. Gresh, J. Mendez-Rico)

**Keywords:** Western equine encephalomyelitis, viruses, outbreak, Uruguay, zoonoses, meningitis/encephalitis

## Abstract

We report the genomic analysis from early equine cases of the Western equine encephalitis virus outbreak during 2023–2024 in Uruguay. Sequences are related to a viral isolate from an outbreak in 1958 in Argentina. A viral origin from South America or continuous enzootic circulation with infrequent spillover is possible.

In November 2023, multiple outbreaks of equine encephalomyelitis were reported in the central Argentina provinces of Corrientes and Santa Fe and then in western Uruguay (Pan American Health Organization, pers. comm., email, 2023 Dec 19). On December 5, 2023, Western equine encephalitis virus (WEEV) was confirmed as the causative agent of an equine death from Salto Department, in northwestern Uruguay (Figure 1). Through March 2024, this outbreak has extended across Uruguay and affected 1,086 equines. We report the diagnosis and preliminary genomic analysis of WEEV on the basis of partial sequencing of the nonstructural protein (NSP) 4 gene that was conducted in the first case of the outbreak (November 28, 2023) and 7 additional cases during December 2023–February 16, 2024. 

**Figure 1:**
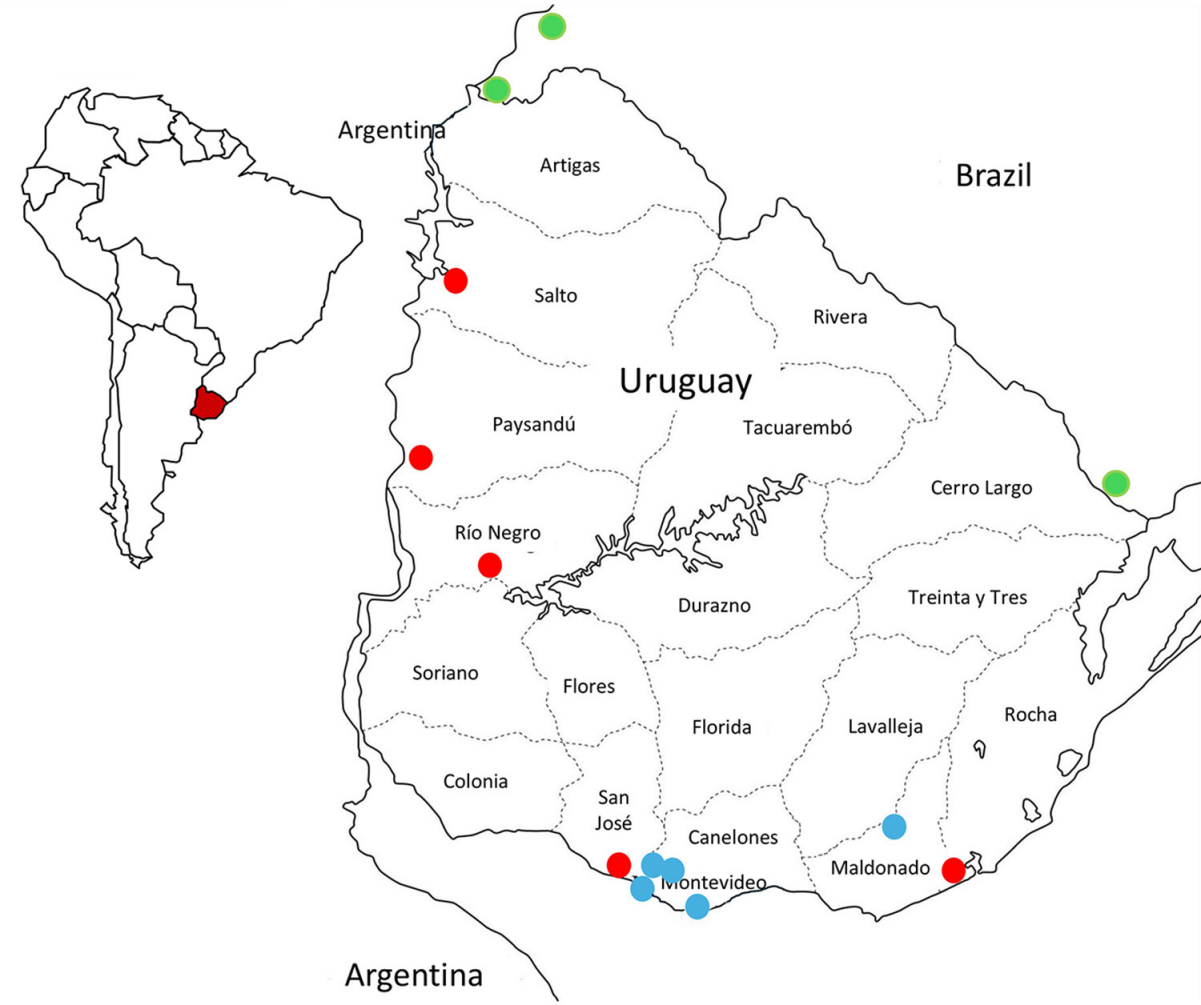
Location of samples analyzed in investigation of equine encephalomyelitis outbreak in Uruguay, 2023–2024. Red dots indicate equine Western equine encephalomyelitis virus cases in Uruguay. Green dots represent sequences retrieved from GenBank that correspond with equine Western equine encephalomyelitis virus cases from Rio Grande do Sul, Brazil. Blue dots represent human cases. Inset map shows location of Uruguay in South America.

We collected equine brain tissue samples from 5 departments: Salto, Paysandú, Rio Negro, San José, and Rocha (Figure 1). We conducted next-generation sequencing (NGS) on 3 of the partially sequenced samples and 3 additional samples by using the Illumina MiniSeq (Illumina, https://www.illumina.com), revealing near-to-complete genomes ranging from 11436 to 11508 nucleotides (Appendix Table). We conducted nucleic acid extraction by using DNA/RNA Pathogen Miniprep Kit (Zymo Research, https://www.zymoresearch.com) or the Tacomini Authomatic Nucleic Acid Extraction System (GeneReach Biotechnology, https://www.genereach.com) on samples and cerebrospinal fluid from dead or symptomatic horses, according to the manufacturer’s instructions. We performed diagnostics by using a generic reverse transcription nested or seminested PCR targeting a phylogenetically informative region of the NSP4 gene (*1*) as modified in previous publications (*2*). This protocol enabled us to accurately identify any member of the alphavirus genus by using Sanger sequencing of the PCR amplicons and further phylogenetic analysis.

Seminested amplicons (303 and 372 bp) were sequenced at Macrogen (Seoul, South Korea) and at the Departamento de Laboratorios de Salud Pública sequencing facility. NGS was performed by using the Viral Surveillance Panel from Illumina (Illumina), which enables whole-genome sequencing of high-impact viruses by using hybrid-capture enrichment. We aligned the sequences obtained with selected alphavirus sequences downloaded from GenBank by using Mafft software (*3*). We reconstructed phylogenies under the maximum likelihood criterion by using PhyML (https://github.com/stephaneguindon/phyml) and midpoint rooting. We calculated branch supports by using the approximate likelihood ratio test and we considered supports > 0.7 as significant (*4*). Phylogenetics trees inferred on NSP4 partial sequences (Figure 2, panel A) or on complete genomes (Figure 2, panel B) showed that sequences from Uruguay form a monophyletic group into the WEEV clade together with sequences from Brazil. The 2023–2024 sequences (Uruguay and Brazil) were closely related to an old virus from Argentina isolated from a sick horse in 1958 in Córdoba (GenBank accession no. KT844543). Also related to the clade from Uruguay are 2 additional sequences from Argentina. The first is an isolate collected in 1933 from a horse from Buenos Aires (accession no. KT844524), and the second isolate is from a *Culex* spp. mosquito collected in 1980 in Chaco province (accession no. GQ287646). The outbreak sequences, together with the old sequences from Argentina, group independently from the North America sequences and do not fall into classifications proposed by previous publications (*5*,*6*). Of note, the sequence from Uruguay retrieved from the 2009 human case (accession no. HM640251.1) (*7*) was unrelated to the sequences recovered from the current outbreak and clusters into the B3 clade with United States sequences (Figure 2). The phylogenies inferred with both partial and complete sequences showed the same overall topology, reinforcing the usefulness of our approach for a sensitive, accurate, and rapid identification of the outbreak’s viral cause.

**Figure 2:**
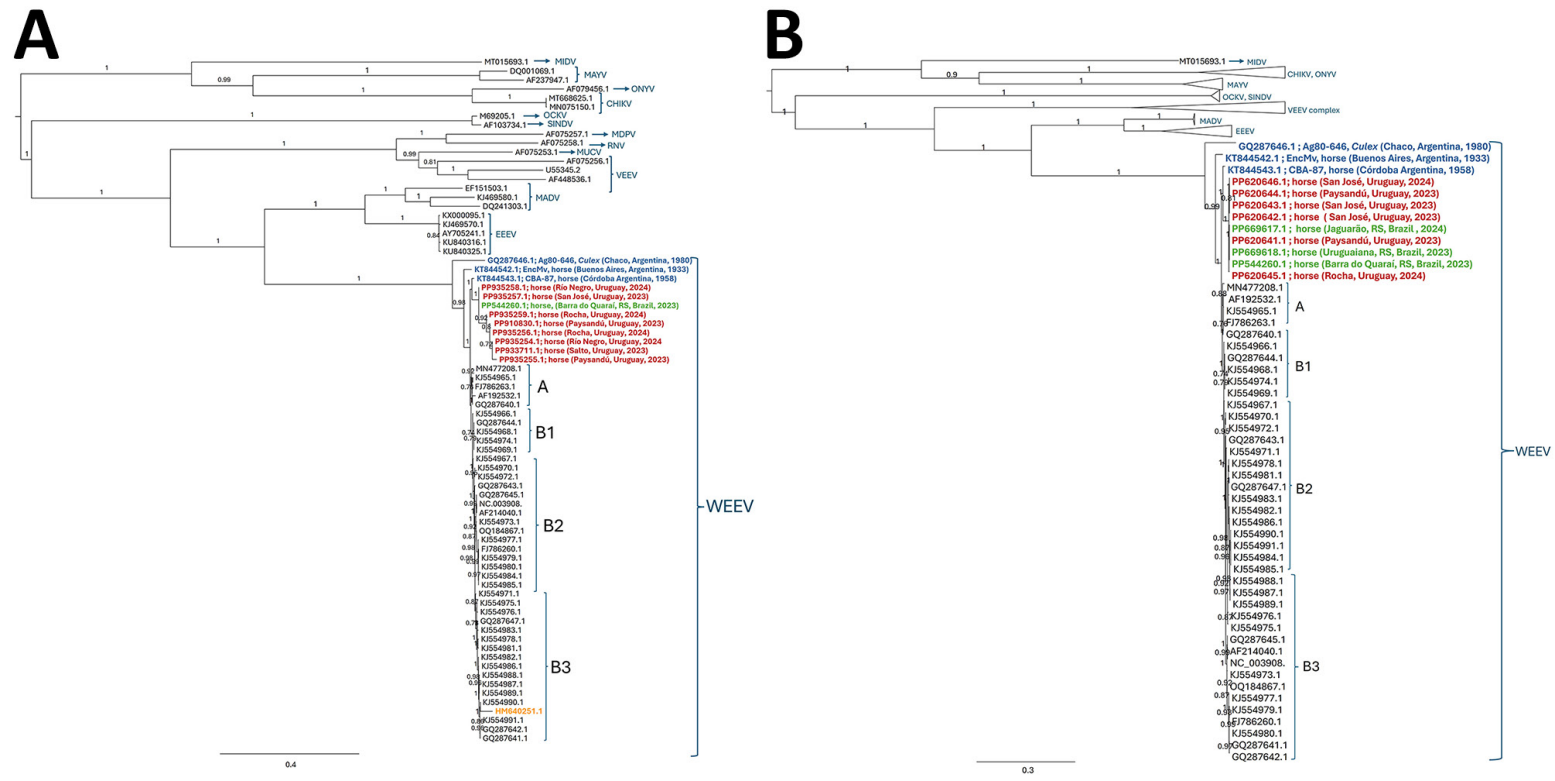
Maximum-likelihood phylogenetic analysis of alphavirus sequences from South and North America and WEEV sequences described in investigation of equine encephalomyelitis outbreak, Uruguay. A) Phylogeny based on partial nonstructural protein 4 gene sequences. B) Phylogeny based on complete sequences. GenBank accession numbers are provided. Subclades are assigned as previously described (*5*,*6*). Clades including reference sequences from other alphaviruses were collapsed for better visualization. Red, sequences from Uruguay 2023–2024; orange, 2009 sequences; blue, sequences from Argentina; green, sequences from Brazil. Branch numbers are approximate likelihood ratio supports. Scale bar indicates substitutions per site. CHIKV, chikungunya virus; EEEV, Eastern equine encephalomyelitis virus; MADV, Madariaga virus; MDPV, Mosso das Pedras virus; MIDV, Middelburg virus; MAYV, Mayaro virus; MUCV, Mucambo virus; OCKV, Ockelbo virus; ONYV, o'nyong-nyong virus; RNV, Rio Negro virus; SINDV, Sindbis virus; VEEV, Venezuelan equine encephalitis virus; WEEV, Western equine encephalomyelitis virus.

In Uruguay, early studies from the 20th Century reported the circulation of several encephalitic alphaviruses in adults and children by using hemagglutination inhibition or complement fixation tests (*8*). More recently, we used a plaque reduction neutralization assay to identify a seropositive horse from a sample collected in 2007 (*9*) and reverse transcription PCR followed by sequencing to diagnose the fatal human case that occurred in 2009 (*7*). In North America, there have been no reports of equine or human WEEV cases since 1998; however, WEEV was detected in mosquito vectors through 2008 (*5*).

The origin and rapid spread of this outbreak are concerning. The positions of the sequences found are related to an old virus strain from Argentina, which might imply the virus remained enzootic in the region for a long period. In addition, a continuous enzootic WEEV circulation in the region, with rare events of spillover to equids and humans, should be considered as a potential origin. A highly rainy spring season and the extensive flooding in 2023 in central Argentina, Uruguay, and southern Brazil were followed by increased mosquito proliferation, especially of the flooding mosquito (*Aedes albifasciatus*), and are likely related to the 2023–2024 outbreak. This set of environmental conditions, characteristics of vertebrate hosts (such as avian species because of their migratory patterns and ecology), and vectors that drove this epizootic outbreak need further investigation under a multidisciplinary approach. Field work is crucial to identifying the vertebrate hosts and the mosquito species acting as WEEV vectors in this region.

Appendix Additional information about equine encephalomyelitis outbreak, Uruguay, 2023–2024.
