# Supplementary material for: Equine Encephalomyelitis Outbreak, Uruguay, 2023–2024
Source: Emerg Infect Dis. 2025 Jan;31(1):180–3. doi: 10.3201/eid3101.240915 (PMC11682807; doi:10.3201/eid3101.240915)
Supplement: Appendix — Additional information about equine encephalomyelitis outbreak, Uruguay, 2023–2024. [file 24-0915-Techapp-s1.pdf]

Article DOI: https://doi.org/10.3201/eid3101.240915

EID cannot ensure accessibility for supplementary materials supplied by authors. Readers who have difficulty accessing supplementary content should contact the authors for assistance.

Equine Encephalomyelitis Outbreak, Uruguay, 2023–2024

Appendix

Appendix Table. Accession numbers for partial and complete sequences reported in this work, and the collection location (Department) for each sample*

Department	Accession no. (Sanger, partial nsp4)	Accession no. (NGS genomes)
Salto	PP933711.1	ND
Paysandú	PP910830.1	PP620641.1
Rio Negro	PP935254.1	ND
Paysandú	PP935255.1	ND
San José	PP935257.1	PP620642.1
Rocha	PP935256.1	PP620645.1
Rio Negro	PP935258.1	ND
Rocha	PP935259.1	ND
Paysandú	ND	PP620644.1
San José	ND	PP620643.1
San José	ND	PP620646.1

*ND, not done; NGS, next-generation sequencing
